# State of World Allergy Report 2008: Allergy and Chronic Respiratory
                    Diseases 

**DOI:** 10.1097/1939-4551-1-S1-S4

**Published:** 2008-06-15

**Authors:** Ruby Pawankar, Carlos E Baena-Cagnani, Jean Bousquet, G Walter Canonica, Alvaro A Cruz, Michael A Kaliner, Bobby Q Lanier, Karen Henley

**Affiliations:** 1World Allergy Organization, 555 East Wells Street, Suite 1100, Milwaukee, WI 53202-3823, USA; 2Department of Otolaryngology, Nippon Medical School, Tokyo, Japan; 3Faculty of Medicine, Catholic University, Cordoba, Argentina; 4Montpellier Hospital, Montpellier, France; 5Ospedale San Martino, University of Genova, Genova, Italy; 6ARIA Scientific Committee, Faculdade de Medicina da Bahia (UFBA), Salvador, Brazil; 7Institute for Asthma and Allergy, Wheaton, MD; 8Fort Worth, TX; 9World Allergy Organization Secretariat, London, UK

**Keywords:** allergy, allergic diseases, prevalence, asthma, prevention, report

## Abstract

It is widely recognized that the incidence of allergies and allergic diseases is
                    on the rise globally. As an international umbrella organization for regional and
                    national allergy and clinical immunology societies, the World Allergy
                    Organization is at the forefront of a combined united effort across nations and
                    organizations to address this global concern by promoting the science of allergy
                    and clinical immunology, and advancing exchange of information.

The World Allergy Organization's State of World Allergy Reports will provide a
                    biennial review of allergic diseases worldwide, consider their medical and
                    socioeconomic contexts, and propose effective approaches to addressing these
                    problems.

In this first State of World Allergy Report 2008, experts from different regions
                    of the world have attempted to define the extent of the global allergy problem,
                    examine recent trends, and provide a framework for the collaboration among world
                    medicine, science, and government agencies that is needed to address the rapidly
                    developing issues associated with allergy and allergic diseases.

## 

What may have begun as a crucial species-protecting immune defense against parasites
                in humans is now ironically responsible for one of the most common maladies,
                allergy, which contributes to massive deficits in quality of life and, in some
                cases, length of life.

Allergy represents an immune programming error. Immunoglobulin E is normally
                protective against many parasites, but can also cause the release of histamine and
                other chemical mediators on exposure to otherwise benign proteins present in
                airborne pollens, molds, animal danders, and food. Does immunoglobulin E confuse the
                complex DNA code in these proteins with those of its primary target, parasites?
                Evolutionary adaptationists argue that the human immune system may be calibrated to
                a certain parasitic load: when that parasite burden decreases as society eliminates
                parasitosis through public health measures, the immune system may become excessively
                sensitive and respond defensively to harmless substances because of a state of
                confusion. Exacerbated by an increased exposure to sensitizing allergens, this
                confusion is manifested through allergic diseases such as asthma,
                rhino-conjunctivitis, atopic eczema, urticaria, and anaphylaxis.

The current scientific consensus is that higher standards of hygiene may deprive the
                developing immune response of important immunologic signals during the period from
                birth to six years, signals that are important for steering the lifelong direction
                of the immune system response. This hygiene hypothesis, Holgate^1 ^argues,
                "best accommodates the link between allergy and social class, the urban to rural
                gradient, infant diet, overuse of antibiotics, and the East to West gradient of
                disease."

Thus, in the zeal to improve standards of health and reduce infectious disease, the
                world may be discovering that improved living conditions along with the rapid
                industrialization of developing nations and a changing world climate present
                opportunities, challenges, and complexities for human health that have never before
                been considered. Allergic diseases are increasing in prevalence worldwide and are
                now the most frequent reasons patients seek medical care. Allergies are also
                becoming more complex, and patients frequently have multiple allergic disorders.
                Even the less severe allergic diseases can have a major adverse effect on the health
                of hundreds of millions of patients and diminish quality of life and work
                productivity. Allergy is a major problem for the 21st century, and this problem is
                predicted to worsen as this century moves forward.

As the incidence of allergy and associated diseases has increased, the number of
                health care professionals trained in the diagnosis and treatment of allergy has
                decreased. As a result, untold numbers of patients go undiagnosed or are
                undertreated. Many developing countries have few or no allergy-trained physicians to
                treat millions of allergy and asthma sufferers, and even in developed countries,
                many highly sophisticated areas have no trained allergists.

The best response to these issues is for the community of world allergists to promote
                better the science of allergy and clinical immunology and the exchange of
                information. The World Allergy Organization is at the forefront of attempts to
                understand, address, and respond to these issues.

In this first State of World Allergy Report, experts from different regions of the
                world have attempted to define the extent of the problem, examine recent trends, and
                provide a framework for the collaboration of world medicine, science, and government
                agencies that is needed to address the rapidly developing issues associated with
                allergy and chronic respiratory disease.

The report opens with a review of the state of allergy and common chronic allergic
                respiratory diseases in the Asia-Pacific region, which hosted the World Allergy
                Congress in Bangkok in December 2007 (where this report was first announced). The
                Asia-Pacific region is also the most populous region of the world and encompasses
                many highly developed nations and numerous emerging economies. A significant
                increase in the prevalence of allergies in the emerging societies of this region is
                anticipated as the social and economic environments change to more industrial and
                postindustrial infrastructures. An overview of the global epidemiology of allergic
                diseases in the following section provides information on world trends and contrasts
                the current situation between developed nations and developing economies. The report
                next outlines the active response to the growing problem of allergic diseases by two
                major world organizations: the World Allergy Organization and the Global Alliance
                against Chronic Respiratory Diseases of the World Health Organization. The report
                concludes with speculation about the changing environmental and social factors that
                are expected to influence the future global pattern of allergic diseases and
                considers the resources that will be needed to manage this burgeoning global
                epidemic. The pharmaceutical industry, in its own response to this growing problem,
                has for many years worked alongside the scientific community to ensure that
                scientific discoveries and innovations are put into practice in the service of
                allergy patients and physicians. Recognizing the importance of this review, members
                of the pharmaceutical sector are supporting the State of World Allergy Report
                through an unrestricted educational grant.

## Reference

1. Holgate ST. The epidemic of allergy and asthma. *Nature*. 1999;
                402(suppl):B2-4.

## Allergy And Asthma: Major Problems In The Asia-Pacific Region

Asthma is a chronic inflammatory disease of the airways that is often associated with
                airway hyperresponsiveness and variable airflow obstruction. Asthma is usually
                reversible either spontaneously or with treatment. Allergic rhinitis is an
                immunoglobulin E-mediated inflammation of the nasal mucosa. Asthma and allergic
                rhinitis are two of the most common chronic respiratory (airway) diseases in
                childhood, but both of these diseases also affect adults. The prevalence of these
                diseases increased substantially in many parts of the world during the 20th century.
                Allergic sensitization, that is, the development of specific immunoglobulin E
                antibodies against common allergens, is an important risk factor for asthma, and
                asthma is often associated with allergic rhinitis.

An estimated 300 million persons worldwide have asthma, approximately 50% of whom
                live in developing countries with limited access to essential drugs; therefore,
                asthma is often poorly controlled in these areas.^1 ^Four hundred million
                persons worldwide have allergic rhinitis.^1,2^

Two large international studies, the International Study of Asthma and Allergies in
                Childhood (ISAAC)^3 ^and the European Community Respiratory Health Survey
                    (ECRHS),^4 ^have studied the prevalence of asthma and allergic rhinitis
                worldwide through the use of standardized questionnaires. Both the ECRHS and ISAAC
                have shown substantial variations in the prevalence of asthma and allergic
                rhinoconjunctivitis across countries and regions.

## Asthma And Allergic Rhinitis: Comorbid Chronic Allergic Respiratory
                Diseases

The ISAAC showed that in general, with some exceptions, higher levels of allergic
                rhinitis or hay fever are observed in communities with higher levels of asthma. Up
                to 70% of persons in the ECRHS who reported having asthma also reported having hay
                fever, and in all centers, hay fever was strongly associated with the presence of
                    asthma.^5 ^Some longitudinal studies suggest that the incidence of
                asthma is more common in those with a history of rhinitis and that the risk is
                greater in those with hay fever of the longest duration and of the greatest severity
                and in those with both sinusitis and rhinitis.^6^

## Asthma And Allergic Rhinitis In The Asia-Pacific Region

### Increasing Prevalence

Asia is the world's most populous continent, with a population of almost 4
                    billion people and many emerging economies. According to recent epidemiological
                    data from ISAAC phase III, asthma and allergic rhinitis have increased in
                    several areas, mostly in low-and middle-income countries.^7,8 ^The
                    prevalence of allergic diseases in Asia varies widely but was found to have
                    increased (from 0.8% to 29.1% for asthma and from 5% to 45% for allergic
                    rhinitis) as communities adopted modern lifestyles and became urbanized.

Time trends in the prevalence of asthma symptoms also showed different regional
                    patterns, that is, a decrease in current wheezing in children aged 13 to 14
                    years in western Europe and an increase in wheezing children of the same age
                    group in the Asia-Pacific region. Even within the Asian region, there was a wide
                    variation among countries. The prevalence of asthma in Japan increased from 3.5%
                    in 1982 to 4.6% in 1992^9 ^to 9.1% in 2006 (A. Akasawa, M.D., Ph.D.,
                    unpublished data, 2006) and was accompanied by an increase in allergic rhinitis
                    of up to 32% (A. Akasawa, M.D., Ph.D., unpublished data, 2006). Similarly, 2
                    surveys performed in Taiwan using an identical method showed that the prevalence
                    of childhood asthma had increased from 1.3% in 1974 to 5.07% in 1985.^10
                    ^More recent ISAAC III data showed that in 13-and 15-yearold children in
                    Taiwan, the overall cumulative and 12-month prevalences of wheezing and rhinitis
                    in the younger children were 8.2% and 44.4%, respectively, and those in the
                    older children were 6.9% and 42.2%, respectively.^11 ^The percentage of
                    children in Singapore who had experienced asthma at least once increased from
                    5.5% in 1967 to 13.7% in 1987 and to 20.7% in 1996. In Singaporean preschoolers
                    aged 4 to 6 years, the cumulative and previous 12-month prevalences of wheezing
                    were 27.5% and 16.0%, respectively. Asthma was reported by 11.7% of this group
                    of children, and the current prevalence of rhinitis was 25.3%.

A field study conducted in four major cities in India with the use of a validated
                    questionnaire showed the overall prevalence of asthma in 2006 to be
                        2.38%.^12 ^In a recent study in rural Bangladesh, the prevalence of
                    asthma in children was 16.1%.^13 ^In contrast, in nonrural Lhasa,
                    Tibet, the prevalences of current wheezing and diagnosed asthma were 0.8% and
                    1.1%, respectively.^14 ^The prevalence of allergic rhinoconjunctivitis
                    in Tibet was 5.2%. Even within the same country, the prevalence of asthma
                    differed among various populations.^7,8 ^Although overall regional data
                    for adults are scant, between 1% and 10% of adults are estimated to have asthma,
                    and between 10% and 32% are estimated to have allergic rhinitis.

### Triggers And Risk Factors

Aeroallergens that trigger allergy and asthma vary from area to area in
                    geographically diverse Asia. Although house-dust mites are the major triggering
                    allergen in most of Asia, pollens, such as Japanese cedar pollen, are a major
                    cause of allergic rhinitis in Japan. In a study of the prevalence of allergen
                    sensitization among asthma patients in Thailand, house-dust mites (both
                        *Dermatophagoides *species and *Blomia
                        tropicalis*) were the most common sensitizing allergens in both
                    pediatric and adult patients with asthma.^15 ^Other important
                    allergens, in order of priority, were cockroach and oil palm pollen. In
                    contrast, less than 5% of patients were sensitive to other pollens and spores.
                    Similarly, in a study of the sensitization profile of the general population in
                    Southeast Asia to house-dust mites, subjects with rhinitis were most sensitive
                    to *B. tropicalis*, followed by *Dermatophagoides
                        pteronyssinus *(73% and 50%, respectively).^16 ^Dual
                    sensitization was common.

Although genetic factors are important in the manifestation of asthma and
                    allergic rhinitis, the rapid increase in the prevalence of these disorders
                    cannot be attributed to genetic factors alone. Changes in environmental factors
                    also need to be taken into account. In a survey that compared the prevalence of
                    asthma and atopic disorders in Chinese children aged 12 to 18 years in three
                    Asian cities (Hong Kong, Kota Kinabalu, and San Bu, with Hong Kong being the
                    most developed and westernized city), the prevalence of asthma and allergic
                    disorders in children from Hong Kong was 2 to 6 times that in children from the
                    other 2 cities.^17 ^Allergic sensitization was a significant factor
                    associated with asthma. The prevalence of atopy in Kota Kinabalu was high (64%),
                    yet the prevalence of asthma was low (1.9%). In a cross-sectional prevalence
                    analysis of wheezing, rhinitis, and eczema in Singaporean preschoolers aged 4 to
                    6 years, the main risk factors for current wheezing and self-reported asthma
                    were family history of allergy, concurrent rhinoconjunctivitis, concurrent
                    chronic flexural rash, and previous respiratory tract infection.^18 ^In
                    rural Bangladesh, risk factors associated with wheezing were pneumonia (at ages
                    0 to 12 months and 13 to 24 months), maternal asthma, paternal asthma, and
                    maternal eczema.^13^

Despite extensive research on genetic, environmental, and lifestyle causes of
                    asthma and on asthma risk factors--including pollution, tobacco smoke, diet,
                    urban lifestyle, reduced early exposure to infections, and viral infections--no
                    single factor has been identified as responsible for the marked geographic
                    variation in or the increasing prevalence of asthma.

### Morbidity and Mortality

Patients with asthma and allergic rhinitis have a reduced quality of life, and
                    the burden of asthma, as assessed by disability-adjusted life-years, ranks 22nd
                    among all diseases worldwide.^19 ^Moreover, asthma in infancy often
                    goes unrecognized and thus untreated.

The Asthma Insights and Reality in Asia-Pacific Study, which looked at patient
                    perceptions of asthma management across Asia, concluded that patients experience
                    frequent and unnecessary symptoms and exacerbations because of a lack of
                    adequate asthma control.^20 ^Indeed, 27% of adults and 37% of children
                    with asthma in the Asia-Pacific region reported that this condition had resulted
                    in an absence from school or work in the previous year, and 40% reported being
                    hospitalized, visiting the emergency department or making unscheduled emergency
                    visits to other health care facilities in the previous year. The severity of
                    asthma varied, with Vietnam and China reporting the most patients with severe
                    persistent symptoms. Work absence was highest in the Philippines (46.6%) and
                    lowest in South Korea (7.5%). In another survey of parents of children with
                    asthma from four Asian countries, most of the children (73%) had preexisting
                    symptoms of allergic rhinitis at the time when asthma was diagnosed, and
                    comorbid asthma and allergic rhinitis substantially affected quality of life and
                    worsened asthma symptoms.^21^

Mortality associated with asthma varies from country to country and seems to be
                    high in countries where access to essential drugs is low. The Global Initiative
                    on Asthma estimates that approximately 250,000 persons die of asthma annually,
                    and the death rate per 100,000 persons with asthma aged 5 to 34 years in highly
                    populated China is greater than 10%.^22 ^However, asthma can be
                    controlled with optimal treatment. This has been proven in countries where an
                    asthma management plan was implemented and the morbidity rate subsequently
                        decreased.^23^

### Socioeconomic Burden

The annual costs of treating asthma and allergic rhinitis--both direct costs
                    (hospitalization, medications) and indirect costs (time lost from work,
                    premature death)--are substantial and represent an even heavier burden in
                    societies with emerging economies. The Asthma Insights and Reality in
                    Asia-Pacific survey of urban centers in eight countries in the Asia-Pacific
                    region showed that the annual per-patient direct costs ranged from US $108 in
                    Malaysia to US $1010 in Hong Kong.^24 ^Total per-patient costs,
                    including productivity costs, ranged from US $184 in Vietnam to US $1189 in Hong
                    Kong. Urgent care costs were 18% to 90% of the total per-patient direct costs.
                    The economic burden in the Asia-Pacific region was higher than that in the
                    United States in relation to the per capita gross domestic product (13% in the
                    Asia-Pacific region compared with 2% in the United States) and per capita health
                    care spending (300% in the Asia-Pacific region compared with 12% in the United
                        States).^24,25 ^Approximately US $20 billion are spent globally
                    each year in relation to allergic rhinitis--a figure that includes the costs
                    associated with medications, lost work productivity, and physician
                    consultations. Approximately US $3 billion are spent in Japan alone.

## Conclusions

The prevalence of chronic allergic respiratory diseases such as asthma and allergic
                rhinitis is increasing in the Asia-Pacific region. With the projected increase in
                the Asian population over the next decade, the burden of allergic diseases is
                expected to increase markedly. The exact mechanisms and determining factors
                underlying the increase in prevalence remain unclear, although allergen
                sensitization has been found to be at least as common in the region as in the West.
                Better identification of triggers and risk factors, increased surveillance of the
                burden of disease, better public awareness, improved training of physicians and
                health care personnel, better access to essential drugs, implementation of
                environmental controls, appropriate management, and implementation of preventive
                    measures^26 ^are key to reducing the burden of these diseases in the
                Asia-Pacific region.

## References

1. Bousquet J, Dahl R, Khaltaev N. Global alliance against chronic respiratory
                diseases. *Allergy*. 2007;62:216-223.

2. Bousquet J, Van Cauwenberge P, Khaltaev N. For the Aria Workshop Group,World
                Health Organization. Allergic rhinitis and its impact on asthma. *J Allergy
                    Clin Immunol*. 2001;108(suppl 1):S147-S334.

3. Asher MI, Montefort S, Björkstén B, Lai CK, Strachan DP, Weiland
                SK, et al. Worldwide time trends in the prevalence of symptoms of asthma, allergic
                rhinoconjunctivitis, and eczema in childhood: ISAAC Phases One and Three repeat
                multicountry cross-sectional surveys. *Lancet*. 2006;368:733-743.

4. Janson C, Anto J, Burney P, Chinn S, de Marco R, Heinrich J, et al. The European
                Community Respiratory Health Survey: what are the main results so far? European
                Community Respiratory Health Survey II. *Eur Respir J*.
                2001;18:598-611.

5. Leynaert B, Neukirch C, Liard R, Bousquet J, Neukirch F. Quality of life in
                allergic rhinitis and asthma. A population-based study of young adults. *Am J
                    Respir Crit Care Med*. 2000;162:1391-1396.

6. Guerra S, Sherrill DL, Martinez FD, Barbee RA. Rhinitis as an independent risk
                factor for adult-onset asthma. *J Allergy Clin Immunol*.
                2002;109:419-425.

7. Björkstén B, Clayton T, Ellwood P, Stewart A, Strachan D. For the
                Phase III Study Group II. World time trends for symptoms of rhinitis and
                conjunctivitis: phase III of the International Study of Asthma and Allergies in
                Childhood. *Pediatr Allergy Immunol*. 2008;19: 110-124.

8. Pearce N, Aït-Khaled N, Beasley R, Mallol J, Keil U, Mitchell E, et al.
                For the ISAAC Phase Three Study Group. Worldwide trends in the prevalence of asthma
                symptoms: phase III of the International Study of Asthma and Allergies in Childhood
                (ISAAC). *Thorax*. 2007;62: 758-766.

9. Nishima S. A study on the prevalence of bronchial asthma in school children in
                western districts of Japan--comparison between the studies in 1982 and in 1992 with
                the same methods and same districts. *Arerugi*. 1993;42:192-204.

10. Hsieh KE, Shen JJ. Prevalence of childhood asthma in Taipei, Taiwan and other
                Asian Pacific countries. *J Asthma*. 1988;25:73-82.

11. Chiang LC, Chen YH, Hsueh KC, Huang JL. Prevalence and severity of symptoms of
                asthma, allergic rhinitis, and eczema in 10 to 15-year-old schoolchildren in central
                Taiwan. *Asian Pac J Allergy Immunol*. 2007; 25:1-5.

12. Aggarwal AN, Chaudhry K, Chhabra SK, D'Souza GA, Gupta D, Jindal SK, et al.
                Asthma Epidemiology Study Group. Prevalence and risk factors for bronchial asthma in
                Indian adults: a multicentre study. *Indian J Chest Dis Allied Sci*.
                2006;48:13-22.

13. Zaman K, Takeuchi H, Md Y, El Arifeen S, Chowdhury HR, Baqui AH, et al. Asthma in
                rural Bangladeshi children. *Indian J Pediatr*. 2007;74:539-543.

14. Droma Y, Kunii O, Yangzom Y, Shan M, Pingzo L, Song P. Prevalence and severity of
                asthma and allergies in schoolchildren in Lhasa, Tibet. *Clin Exp
                    Allergy*. 2007;37:1326-1333.

15. Yeoh SM, Kuo IC, Wang DY, Liam CK, Sam CK, De Bruyne JA, et al. Dermatophagoides
                pteronyssinus and Blomia tropicalis. Sensitization profiles of Malaysian and
                Singaporean subjects to allergens. *Int Arch Allergy Immunol*.
                2003;132:215-220.

16. Leung R, Ho P. Asthma, allergy, and atopy in three south-east Asian populations.
                    *Thorax*. 1994;49:1205-1210.

17. Daengsuwan T, Lee BW, Visitsuntorn N, Charoenratanakul S, Ruangrak S,
                Jirapongsananuruk O, et al. Allergen sensitization to aeroallergens including Blomia
                tropicalis among adult and childhood asthmatics in Thailand. *Asian Pac J
                    Allergy Immunol*. 2003;21: 199-204.

18. Tan TN, Shek LP, Goh DY, Chew FT, Lee BW. Prevalence of asthma and comorbid
                allergy symptoms in Singaporean preschoolers. *Asian Pac J Allergy
                    Immunol*. 2006;24:175-182.

19. World Health Organization. *The World Health Report 2004: Changing
                    History*. Geneva, Switzerland: World Health Organization; 2004.

20. Zainudin BM, Lai CK, Soriano JB, Jia-Horng W, De Guia TS, For the Asthma Insights
                and Reality in Asia-Pacific (AIRIAP) Steering Committee. Asthma control in adults in
                Asia-Pacific. *Respirology*. 2005;10:579-586.

21. Valovirta E, Pawankar R. Survey on the impact of comorbid allergic rhinitis in
                patients with asthma. *BMC Pulm Med*. 2006;30(suppl 1):S3.

22. The Global Initiative for Asthma. Available at: http://www.ginasthma.com.
                Accessed April 24, 2008.

23. Haahtela T, Klaukka T, Koskela K, Erhola M, Laitinen LA. For the Working Group of
                the Asthma Programme in Finland 1994-2004. Asthma programme in Finland: a community
                problem needs community solutions. *Thorax*. 2001;56:806-814.

24. Lai CKW, Kim Y-Y, Kuo S-H, Spencer M, Williams AE. Asthma Insights and Reality in
                Asia Pacific Steering Committee. Cost of asthma in the Asia-Pacific region.
                    *Eur Respir Rev*. 2006;98:10-16.

25. Bateman ED. The economic burden of uncontrolled asthma across Europe and the
                Asia-Pacific region: can we afford to not control asthma? *Eur Respir
                    Rev*. 2006;15:1-3.

26. Weiss K, Haus M, Likura Y. The costs of allergy and asthma and the potential
                benefit of prevention strategies. In: Johansson SGO, Haahtela T, eds.
                    *Prevention of allergy and allergic asthma. World Allergy Organization
                    report and guidelines. Chemical Immunology and Allergy*, vol 84. Basel,
                Switzerland: Karger; 2004:184-192.

## The Worldwide Increase In Allergy And Asthma

The global prevalence of asthma and the morbidity, mortality, and economic burden
                associated with it have increased sharply over the past 40 years, particularly in
                children. The prevalence of asthma has increased by 50% every decade. In North
                America, an estimated 22.2 million persons (6.5 million children and 15.7 million
                adults) have asthma, and in 2003, asthma accounted for 1.4 deaths per 100,000
                    persons.^1 ^Asthma is underdiagnosed and under-treated, although the
                use of inhaled corticosteroids has had a positive impact on outcomes.

The increasing number of hospital admissions for asthma, which are most pronounced in
                young children, reflects an increase in asthma severity in part because of poor
                disease management. Worldwide, approximately 250,000 deaths per year are
                attributable to asthma, although overall mortality rates have decreased since the
                1980s. Most asthma deaths occur in those aged 45 years and older and are largely
                preventable. Death is frequently related to inadequate long-term medical care
                (including the lack of affordability in low-income groups) or to delays in obtaining
                medical help during an attack.^2^

The financial burden related to asthma in different western countries ranges from US
                $300 to US $1300 per patient per year and disproportionately affects those with the
                most severe disease.^3 ^The burden in weaker economies is even greater.
                Several significant barriers exist to reducing the burden of asthma, particularly in
                low-income groups, in whom access to care and essential medications is limited.

Contrary to the global increases seen in recent decades, some reports have shown no
                change in asthma trends over the past decade. Such discrepancies exist even among
                studies from the same country. In addition to differences in methods and the age
                distribution of the population studied, differences in the degree of urbanization
                and in the occurrence and magnitude of protective (yet only partially identified)
                factors among the study areas may explain these discrepancies. Furthermore,
                geoclimatic variables and topographic factors, for example, the altitude at which
                people live, may also affect the prevalence of asthma.

Accumulating evidence from some countries, particularly developed countries,
                indicates that the rising trends in asthma prevalence among adults may now have
                plateaued or even decreased. The data are more contradictory in children. Several
                studies have shown stable trends in childhood asthma since the late 1990s, whereas
                others have not shown any change in the steadily rising asthma trends. In contrast,
                an upward trend in the prevalence of asthma has been described in those countries
                with a previously low prevalence, mainly in developing countries.^4
                ^However, nearly all of these studies lack the most recent data from the 2000s.
                Moreover, environmental and lifestyle factors may continue to induce asthma symptoms
                in susceptible persons until the saturation level in prevalence, determined by the
                genetic composition of the population, is achieved. Defects in environmental Th1
                triggering and in inborn Th1 maturation are examples of the environment-and
                gene-associated factors that might influence asthma prevalence.^5 ^However,
                severalfold higher peak prevalence rates have been reported in Australia, and no
                peak prevalence rates have yet been reported in many countries with high rates of
                asthma. Because nearly all the studies referred to here were conducted in affluent
                westernized countries, no consideration of the results in light of the East-West
                gradient could be performed. It remains to be determined whether the increased use
                of antibiotics is involved in the stabilization or decrease observed, particularly
                in the prevalence of non-immunoglobulin E (IgE)-mediated (sometimes termed
                nonatopic) adult asthma, for which an infectious etiology has been proposed.^6
                ^The results of long-term follow-up, derived from large multinational surveys,
                including the European Community Respiratory Health Survey and International Study
                of Asthma and Allergies in Childhood (ISAAC), will be a good indicator of current
                asthma trends on a global scale.

The Global Initiative for Asthma^7 ^has outlined a 6-point patient
                management plan to address the effective handling of the increased number of asthma
                patients receiving primary care. The plan focuses on patient education, written
                treatment plans, and ongoing communication and review between patients and health
                care providers. Although the asthma prevention and management programs have
                undoubtedly contributed favorably to the reduction in asthma prevalence in many
                countries, the possibility that asthma has become milder, independently of the
                increased use of inhaled corticosteroids, cannot be excluded. Furthermore, few
                studies have analyzed time trends in the prevalence of IgE-mediated and
                non-IgE-mediated asthma separately. Limited data from the United Kingdom and Finland
                indicate that the prevalence of IgE-mediated allergic asthma has continued to
                increase over the past few decades, whereas that of nonYIgE-mediated asthma has not.
                This may indicate that the proportions of IgE-mediated and nonYIgE-mediated asthma
                within the overall asthma burden are also changing, which may additionally explain
                the finding that asthma has become milder. In the Finnish young male population, the
                prevalence of asthma increased from 0.29% in 1966 to 1.79% in 1989.^8 ^In
                the United Kingdom, an evaluation of the prevalence of asthma in schoolchildren
                between 1991 and 2002 showed a significant increase in wheezing in the past 12
                months and in severe speech-limiting episodes and night waking, but no significant
                increase in medical visits because of wheezing.^9 ^Another time trend study
                also showed a significant increase in physician-diagnosed asthma from 1990 to 2003,
                which was more evident in females (range, 7.3%-14.6%) than in males (range,
                7.8%-9.4%) in all age groups, particularly in persons aged 55 years and
                    older.^10^

## Prevalence Of Allergy And Asthma In Low-And Middle-Income Countries

In a study that compared the prevalence of asthma and atopy between children from
                affluent countries and children from nonaffluent countries, the prevalence of
                wheezing and persistent cough was lower in Nigeria than in Australia (10.2% and 5.1%
                compared with 21.9% and 9.6%, respectively).^11 ^A recent report from
                Ait-Khaled et al^12 ^showed a wide range of atopic disorders that were
                prevalent throughout Africa, with the highest prevalence of current asthma in urban
                areas with a higher standard of living (in agreement with the hygiene hypothesis).
                However, the prevalence of asthma was also high in endemic parasite and tuberculosis
                zones (in opposition to the hygiene hypothesis).^12 ^In Latin America, the
                prevalence of asthma and allergic diseases in childhood was similar to that in
                industrialized countries, although the variability was great.^13 ^In a
                recent survey conducted in Asia, the prevalence of wheezing in rural children from
                Bangladesh over the previous 12 months was 16%, which was not significantly
                different from the prevalence observed in other developing regions.^14
                ^Taken together, the evidence shows that the prevalence of asthma is high and
                is still increasing slightly, mainly in developing countries.

## Overcoming Barriers: A Global Perspective With Emphasis On Isaac

The ISAAC is a unique global epidemiological study of the prevalence and severity of
                asthma, rhinitis, and eczema,^15 ^although other studies of trends in the
                prevalence of symptoms of asthma, allergic rhinoconjunctivitis and eczema in
                children and adolescents have used similar methods. In the counties of Troms and
                Finnmark in Norway, divergent trends in symptom prevalence were observed.
                Investigators reported no change in the prevalence of symptoms of asthma, hay fever,
                or eczema in centers in eastern and southwestern Germany, whereas other
                investigators reported an increase in the prevalence of hay fever symptoms, but not
                that of asthma or eczema, in East Germany. In Ankara, Turkey, a significant decrease
                in the cumulative prevalence of allergic rhinitis was noted, with a slight
                insignificant increase in the prevalence of asthma and eczema. Tallinn in Estonia
                was the only center included in the present study for which trends for asthma,
                allergic rhinoconjunctivitis, and eczema had been studied previously. Little change
                was reported in the prevalence of wheezing, rhinitis, and itching rash between
                cross-sectional studies completed in 1992-1993 and 1996-1997. In Aberdeen, United
                Kingdom, the prevalence of asthma symptoms plateaued,^16 ^whereas the
                prevalence of eczema and hay fever symptoms increased.

The data on trends in symptoms of asthma, allergic rhinoconjunctivitis, and eczema
                have direct relevance to health service delivery in the countries included in the
                studies and provide a basis for understanding these disorders. In almost all
                participating centers, the prevalence of one or more of the disorders changed over
                time. Although changes in the mean annual prevalence of approximately 0.5% might
                sound small, such changes could have substantial public health implications,
                especially because the increases took place primarily in heavily populated
                countries. Urban centers in developing countries might have few resources to
                implement management programs for these diseases in the face of an overwhelming
                number of infectious diseases. The level of interest from centers in developing
                countries participating in prevalence studies indicates a concern that asthma and
                allergies in children are emerging as important public health
                problems.^4^

The ISAAC has provided valuable data on the prevalence and severity of asthma,
                rhinitis, and eczema. The prevalence of asthma is highest in English-speaking
                countries, and little variation exists within English-speaking countries. The
                overall prevalence is higher than expected, particularly in Latin America. However,
                variation exists among and within countries; for example, there is a
                northwest-southwest gradient within Europe. The correlation between asthma
                prevalence and the gross national product is inconsistent. In some areas (eg,
                Germany and China), a major difference in prevalence exists within the same ethnic
                group, and a weak and inconsistent association exists between the prevalence of
                asthma and that of other allergic conditions. The ISAAC phase III data suggest an
                upward trend in the prevalence of asthma in Argentina. In Cordoba, the only center
                that participated in both phases I and III, the prevalence increased from 11.2% to
                11.6%.

In conclusion, prevalence studies, such as ISAAC, that take into account
                differentials in variables such as poverty, population, immigration patterns, and
                ethnic and cultural differences are a major step toward overcoming existing barriers
                to the worldwide diagnosis and treatment of asthma.

## References

1. National Center for Health Statistics. Asthma prevalence, health care use and
                mortality: United States, 2003Y2005. Available at: http://www.cdc.gov/nchs/products/pubs/pubd/hestats/ashtma03-05/asthma03-05.htm#1.
                Accessed October 2007.

2. Masoli M, Fabian D, Holt S, Beasley R. Global Initiative for Asthma (GINA)
                Program. The global burden of asthma: executive summary of the GINA Dissemination
                Committee report. *Allergy*. 2004;59: 469-478.

3. Accordini S, Corsico A, Cerveri I, Gislason D, Gulsvik A, Janson C, et al. Therapy
                and Health Economics Working Group of the European Community Respiratory Health
                Survey II. The socio-economic burden of asthma is substantial in Europe.
                    *Allergy*. 2008;63:116-124.

4. Asher MI, Montefort S, Björkstén B, Lai CK, Strachan DP, Weiland
                SK, et al. ISAAC Phase Three Study Group. Worldwide time trends in the prevalence of
                symptoms of asthma, allergic rhinoconjunctivitis, and eczema in childhood: ISAAC
                Phases One and Three repeat multicountry cross-sectional surveys.
                    *Lancet*. 2006;368:733-743.

5. Romagnani S. Regulatory T cells: which role in the pathogenesis and treatment of
                allergic disorders? *Allergy*. 2006;61:3-14.

6. von Mutius E. Of attraction and rejection-- asthma and the microbial world.
                    *N Engl J Med*. 2007;357:1545-1547.

7. The Global Initiative for Asthma. Available at: http://www.ginasthma.com.
                Accessed April 24, 2008.

8. von Hertzen L, Haahtela T. Signs of reversing trends in prevalence of asthma.
                    *Allergy*. 2005;60:283-292.

9. Butland BK, Strachan DP, Crawley-Boevey EE, Anderson HR. Childhood asthma in South
                London: trends in prevalence and use of medical services. *Thorax*.
                2006;61:383-387.

10. Wilson DH, Adams RJ, Tucker G, Appleton S, Taylor AW, Ruffin RE. Trends in asthma
                prevalence and population changes in South Australia, 1990-2003. *Med J
                    Aust*. 2006;184:226-229.

11. Faniran AO, Peat JK, Woolcock AJ. Prevalence of atopy, asthma symptoms and
                diagnosis, and the management of asthma: comparison of an affluent and a
                non-affluent country. *Thorax*. 1999;54:606-610.

12. Ait-Khaled N, Odhiambo J, Pearce N, Adjoh KS, Maesano IA, Benhabyles B, et al.
                Prevalence of symptoms of asthma, rhinitis and eczema in 13-to 14-year-old children
                in Africa: the International Study of Asthma and Allergies in Childhood Phase III.
                    *Allergy*. 2007;62: 247-258.

13. Mallol J, Solé D, Asher I, Clayton T, Stein R, Soto-Quiroz M. Prevalence
                of asthma symptoms in Latin America: the International Study of Asthma and Allergies
                in Childhood (ISAAC). *Pediatr Pulmonol*. 2000;30: 439-444.

14. Zaman K, Takeuchi H, Md Y, El Arifeen S, Chowdhury HR, Baqui AH, et al. Asthma in
                rural Bangladeshi children. *Indian J Pediatr*. 2007;74: 539-543.

15. ISAAC Home. Available at: http://isaac.auckland.ac.nz/. Accessed April 24, 2008.

16. Ninan TK, Russell G. Respiratory symptoms and atopy in Aberdeen schoolchildren:
                evidence from two surveys 25 years apart. *BMJ*. 1992;
                304:873-875.

## How Organizations Are Responding To The Global Increase In Allergic Respiratory
                Disease

The incidence of chronic allergic respiratory diseases is increasing, and a united
                effort across nations and organizations is needed to address effectively this global
                concern.

## The Global Alliance Against Chronic Respiratory Diseases

The Global Alliance against Chronic Respiratory Diseases (GARD) is a voluntary
                alliance of 70 national and international organizations, institutions, and agencies
                working toward the common goal of reducing the incidence of chronic respiratory
                diseases worldwide, according to local needs.^1 ^The GARD's vision is "a
                world where all people breathe freely."^2 ^The key aim of GARD is to
                initiate a comprehensive approach to fighting chronic respiratory diseases. The GARD
                was created in recognition of the impact that chronic respiratory diseases have on
                the global community. Many chronic respiratory diseases, including asthma and
                rhinitis, are increasing in prevalence, even though these diseases are allergic in
                origin and are therefore preventable.

The 53rd World Health Assembly recognized the enormous human suffering caused by
                chronic diseases and requested the Director General of the World Health Organization
                (WHO) to give priority to the prevention and control of these diseases (WHO
                resolution 53.17, May 2000; endorsed by all 191 WHO Member States). This led to the
                formation of GARD.

The GARD is being developed in a stepwise approach using three planning steps that
                follow the steps outlined in "Preventing Chronic Disease: A Vital Investment," a
                report issued by the WHO. To pursue the vision of "a world where all people breathe
                freely," GARD's mission is to develop an environment that enables sustainable
                appropriate action at individual, community, national, and global levels. The goal
                of all GARD's actions is to reduce the chronic respiratory disease burden
                    globally.^3^

The GARD's specific objectives include the following:

• Developing a standard way of obtaining relevant data on the risk factors
                for and disease burden of chronic respiratory diseases.

• Advocating policies that promote health and disease prevention in countries
                worldwide.

• Developing simple and affordable strategies for the diagnosis and
                management of chronic respiratory diseases in all countries.

• Adapting and tailoring all recommendations to each country's health
                priorities, health care system, diversity of chronic respiratory diseases,
                availability of health care personnel, facilities for diagnosis, and availability
                and affordability of medications.

In addition, GARD aims to improve coordination between existing governmental and
                nongovernmental programs and to avoid duplication of effort and wastefulness of
                resources. Such coordination will also enable participant organizations to work
                synergistically to achieve GARD's objectives.

## Gard Working Groups

Following a model of collaboration and coordinated activity, GARD and the World
                Allergy Organization (WAO) are working together through established GARD working
                groups on major programs in the following four areas: prevention, diagnostics,
                management, and pediatrics.

### Working Group on Health Promotion and Prevention of Chronic Respiratory
                    Disease and Allergies

Everyone has the right to live and work in a clean environment. Exposure to an
                    unhealthy environment can cause severe and debilitating chronic obstructive
                    pulmonary disease, asthma, cardiovascular diseases, and cancer. Complete
                    elimination of risk factors is the only way to remove the risk, and this
                    strategy applies equally to tobacco smoke, indoor and outdoor air pollutants,
                    occupational hazards, and allergens. This working group will encourage countries
                    to implement policies to reduce the burden of tobacco smoke, indoor and outdoor
                    pollution, occupational hazards and other risk factors relevant to chronic
                    respiratory disease.

### Working Group on Diagnosis of Chronic Respiratory Disease and
                    Allergies

The first initiative for the Diagnostics Working Group is to survey WAO Member
                    Societies (national and regional allergy and clinical immunology societies
                    worldwide) to define the most relevant allergens in each country. This will
                    enable the group to ensure the development of low-cost tests for immunoglobulin
                    E sensitization against the most common respiratory and food allergens in any
                    country or region. A second survey was created to obtain information about which
                    in vivo and in vitro tests are most commonly used worldwide to detect
                    respiratory, food, and drug allergies.

The Diagnostics Working Group will also conduct allergy skin prick tests for
                    immunoglobulin E sensitization using either standardized manufactured allergens
                    (in high-and middle-income countries) or locally produced reliable extracts (in
                    middle- and low-income countries). Central to this is the detection of relevant
                    regional allergens, done in cooperation with the WAO Emerging Societies Program
                    (see Developing the Specialty of Allergy Worldwide), which has placed pollen
                    traps in countries where major airborne allergens need to be identified.

### Working Group on Control of Chronic Respiratory Disease and Allergies

The GARD action plans should be tailored to each country's needs, priorities,
                    health services, and resources.

• In areas with a high burden of communicable diseases and a functioning
                    primary health care service, an integrated approach to prevention, diagnosis,
                    and management is recommended. Models such as the WHO-Practical Approach to Lung
                    Health will be promoted.

• In areas with a high prevalence of human immunodeficiency virus
                    infection, models such as PALSA Plus (PAL in South Africa) will be promoted.

• Prevention and care for chronic respiratory disease in middle- and
                    high-income countries may involve the use of different models. Disease-specific
                    approaches can be more relevant and might target asthma, rhinitis, chronic
                    obstructive pulmonary disease, and occupational lung diseases. Approaches will
                    be developed from available management plans and international guidelines
                    according to country-specific needs.

### Working Group on Pediatric Chronic Respiratory Disease and Allergies

Approximately 70% to 80% of asthma in children is allergic in origin, and 5% to
                    15% of the global pediatric population is thought to have asthma.^4
                    ^The Pediatrics Working Group believes that future increases in asthma
                    prevalence will likely be greatest in the developing world. The group has
                    outlined objectives for the diagnosis and management of pediatric asthma in
                    high-, middle-, and low-income countries. The priorities are to develop an
                    asthma-symptom algorithm to aid health care practitioners in making diagnoses
                    and to provide "Is it asthma?" symptom cards, developed by GARD, to facilitate
                    such diagnoses. Pamphlets and posters will also be developed to increase
                    awareness about asthma. Emphasis will continue to be placed on the importance of
                    avoiding tobacco smoke. Finally, prevalence studies will be conducted in
                    preschool children.

## The World Allergy Organizaton

The WAO is an international umbrella organization with a membership consisting of 77
                regional and national allergology and clinical immunology societies from around the
                    world.^5 ^The WAO is a founding member of GARD and is also one of
                GARD's most active and supportive members. The WAO's approach is complementary to
                that of GARD: to fight allergic diseases worldwide. The WAO is in a unique position
                to synthesize the best allergy practices developed by long-established allergy
                societies, to disseminate the knowledge gained throughout diverse geographic
                regions, and to share relevant experiences with newer allergy and clinical
                immunology societies and allergists worldwide. In collaboration with WAO's member
                societies, WAO provides direct educational outreach programs, specialty development
                programs, symposia, and lectureships to members in 92 countries. Chronic allergic
                respiratory disease, which encompasses allergic asthma, rhinoconjunctivitis, and
                sinusitis, is a natural focus of interest for WAO members and thus was selected as
                the focus of the joint WAO/GARD World Allergy Day 2007.

## Defining The Specialty Of Allergy And The Need For Allergy Services

No studies that provide a complete picture of the size of the global allergy epidemic
                have been reported. Figures available from peer-reviewed studies provide only
                limited snapshots of the prevalence of particular allergic diseases in specific
                population subgroups at the time of sampling. In 2005, the WAO Specialty and
                Training Council conducted an informal survey of WAO member societies and requested
                estimates of the proportion of the population in each country with allergic disease
                and estimates of the resources available to manage these patients. The results,
                published in 2006,^6 ^included responses from member societies representing
                33 countries and suggested that approximately 16,000 trained allergists serve an
                estimated total population of 1.3 billion persons, approximately 22% of whom have
                some form of allergic disease. The survey confirmed that most allergy patients are
                not seen by a trained allergist; therefore, these patients may not be receiving
                optimal care. A formal follow-up survey requested information on the documented
                prevalence of asthma, allergic rhinitis, atopic eczema, food allergy, drug allergy,
                and Hymenoptera allergy,^7 ^and provides a clearer picture of the global
                incidence of specific allergic diseases. Studies of anaphylaxis prevalence and
                anaphylaxis management practices are in progress and will be based on international
                surveys being conducted by WAO member societies over the 3-year period from 2007 to
                2009.

The findings from the Allergy Practice Worldwide Survey guided the development of a
                WAO position statement, "Requirements for Physician Competencies in Allergy: Key
                Clinical Competencies Appropriate for the Care of Patients with Allergic or
                Immunologic Diseases--A Position Statement of the World Allergy Organization."^8
                ^This statement provides advice for the development of allergy specialist
                programs and recommends that appropriately targeted educational programs be
                developed for the thousands of nonallergists worldwide who treat allergy
                patients.

The new WAO position statement, "What Is an Allergist?" was published in *The
                    World Allergy Organization Journal *in January 2008.^9 ^This
                position statement outlines what patients and health care providers should expect
                from allergy specialists and aims to help physicians refer patients appropriately to
                allergists. Further statements on recommendations for training on allergies, at both
                the undergraduate level and for pediatric allergists, will be developed during 2008.
                The WAO position statements have been created to establish and strengthen the
                specialty of allergy and to convince governments and health care systems of the
                importance of providing and supporting allergy services.

## Developing The Specialty Of Allergy Worldwide

### The WAO Emerging Societies Program

The WAO Emerging Societies Program encourages the development of new national
                    allergy societies throughout the world, particularly in low-income countries.
                    The program is provided in collaboration with the American College of Allergy,
                    Asthma, and Immunology. While enabling the sharing of practical experiences and
                    alerting aspiring societies to the criteria required for WAO membership, the
                    program helps to create local support networks for allergists through
                    partnerships with established allergy societies within the same region and
                    fosters a relationship with WAO. At the same time, the WAO leadership learns
                    about the challenges and opportunities faced by colleagues in developing
                    countries. Membership in WAO enables fledgling societies to become a part of the
                    global allergy community and to have a voice in WAO's House of Delegates, where
                    the societies can help to determine the organization's future activities.

The WAO is planning a network of local summer schools that will provide hands-on
                    training in allergy diagnostic techniques and methods of treatment delivery in
                    areas where allergy services are newly established. In 2008, the Emerging
                    Societies Program will hold a summer school for primary care physicians in Latin
                    America, an area where allergy services are deficient. The summer school will
                    provide training in the fundamentals of allergy diagnosis and treatment.

The Emerging Societies Program has loaned pollen traps to centers in Pakistan,
                    Ukraine, and Georgia. These traps are helping allergists identify the major
                    local airborne pollens and molds responsible for allergic respiratory diseases
                    in these countries, and the hope is that the information acquired will
                    eventually lead to the availability of appropriately directed diagnostic
                    materials and immunotherapy vaccines. The distribution of more pollen traps is
                    an urgent priority.

### Allergy Learning and Educational Programs

The WAO offers a variety of educational and learning programs that reflect the
                    diversity of learning needs within the global allergy environment. The Global
                    Resources in Allergy (GLORIA) program provides educational materials in the form
                    of didactic lectures on major topics in allergy; these lectures are available on
                    the WAO Web site so that the organization's 35,000 members can use the GLORIA
                    materials in teaching programs.^10 ^The Seminars and Conferences
                    Program provides invited lecturers to speak at member societies' meetings on
                    topics selected to specifically meet the learning needs of the societies' local
                    clinical and scientific allergy community. In 2007, WAO provided lectures on
                    various aspects of allergy to more than 25 allergy and clinical immunology
                    societies throughout the world and will provide even more in 2008.

To further improve education worldwide, WAO has just created an online clinical
                    journal, *The World Allergy Organization Journal*, to be a major
                    academic resource for the world's allergy community.^11^

Focusing on the knowledge objectives for physicians as defined by the Specialty
                    and Training Council,^8 ^WAO is also developing learning programs aimed
                    at meeting the learning needs of the diverse range of physicians who care for
                    patients with allergic diseases. A major strategy of this goal is online
                    education, which is increasingly accessible worldwide and is a cost-effective
                    way of providing learning and educational materials asynchronously at a
                    convenient time for global users. New initiatives for 2008 include a series of
                    11 online lectures on the basic immunology underlying allergic disease and a
                    series of online learning programs based on complex clinical case histories,
                    through which users are guided through the wealth of educational materials that
                    are available on the WAO Web site.

### Promoting Allergy Research

The WAO Research Council awards annual short- and long-term research fellowships
                    enabling junior clinicians to visit an international expert center to learn a
                    clinical or scientific technique that is not available at the home institution
                    or to participate in a research study. These programs aim to help the students'
                    home institutions establish new research programs, and particular emphasis is
                    given to encouraging research in low-income countries. Fellowships are directed
                    toward three broad research priorities:

• Genetic factors involved in the development of allergic disease and
                    response to treatment;

• Allergen characterization and standardization; and

• Clinical and basic studies in allergy and asthma.

### Best-Practice Recommendations and Position Statements

The WAO collaborated with the WHO to develop recommendations for the prevention
                    of allergy and allergic asthma based on the recommendations of a group of
                    international allergy experts representing all WHO regions. The document,
                    published in 2004, summarized current knowledge on the prevention of allergy and
                    allergy-related asthma and made recommendations for future research to address
                    knowledge gaps.^4^

The WAO Research Council oversees the creation of position statements on major
                    topics in allergy. "Recommendations for Standardization of Clinical Trials With
                    Allergen Specific Immunotherapy for Respiratory Allergy: A Statement of a World
                    Allergy Organization Taskforce" was published in 2007 to ensure that new
                    clinical trials on allergen immunotherapy are conducted in a standardized way
                    that will enable worldwide comparability of results.^12^

The WAO is conducting studies on the current availability of epinephrine and on
                    the local management of anaphylaxis in all member society countries. In parallel
                    with these studies, the organization is developing global recommendations on the
                    use of epinephrine for the treatment of anaphylaxis. Although epinephrine is the
                    drug of choice for the treatment of anaphylaxis, this drug is not yet globally
                    available. The hope is that WAO initiatives will assist national societies in
                    persuading health care funding bodies of the importance of making epinephrine
                    widely available for the treatment of anaphylaxis.

### Communication

The WAO Web site (http://www.worldallergy.org) is a major tool for disseminating
                    education and information. A monthly e-newsletter reaches more than 26,000
                    allergists and is available in eight languages. The newsletter provides reviews
                    on current scientific literature and books, updates on WAO activities, and
                    opportunities for member societies to apply for educational programs and
                    fellowships. The WAO Web site contains extensive educational resources and
                    contact information and provides links to all WAO member societies' Web sites
                    and selected allergy resources on the World Wide Web.

World Allergy Day takes place every two years and is themed on activities at the
                    biennial World Allergy Congress. This event provides an opportunity for wider
                    dissemination of knowledge on major topics in allergic disease, with
                    participating member societies, local media, and patient groups focusing on
                    those aspects of allergic disease that have the greatest impact on local
                    communities.

All WAO resources and services are designed to increase the knowledge and
                    awareness of allergic disease and enhance the clinical practice of allergy. The
                    WAO's role is to support member societies in promoting the specialty at a
                    national level and to provide information to help in negotiations to obtain
                    optimal resources for the care of allergy patients. Thus, development of the
                    specialty of allergy, physician education and information, best-practice
                    recommendations, promotion of allergy research, and creation of an awareness of
                    the global allergy burden are critical responsibilities of WAO. Because of the
                    enormous global burden of allergic disease, most patients with allergy will not
                    be seen by an allergist; therefore, non-allergists must be educated to
                    recognize, diagnose, and treat patients with uncomplicated allergy and to know
                    when and how to refer patients with complex disease to an allergist.

## Summary

At a global level, governments and those who fund health care must be informed about
                the burgeoning prevalence of allergic disorders and the likelihood that patient
                numbers will continue to increase. Collaboration between GARD and WAO is of major
                importance to both organizations in fighting the global problem of chronic allergic
                respiratory diseases.

## References

1. World Health Organization. Global Alliance Against Chronic Respiratory Diseases
                Web site. Available at: http://www.who.int/gard.
                Accessed April 24, 2008.

2. Bousquet J, Dahl R, Khaltaev N. Global alliance against chronic respiratory
                diseases. *Allergy*. 2007;62:216-223.

3. Bousquet J, Khaltaev N. Global surveillance, prevention and control of chronic
                respiratory siseases. A comprehensive approach. Geneva, Switzerland: Global Alliance
                Against Chronic Respiratory Diseases, World Health Organization; 2007.

4. Johansson SGO, Haahtela T. Prevention of allergy and allergic asthma. In: Ring J,
                et al, eds. Chemical immunology and allergy, vol 84. Basel, Switzerland: Karger;
                2004. Summary available at: http://www.worldallergy.org/professional/who_paa2003.pdf. Accessed
                April 23, 2008.

5. World Allergy Organization Web site. Available at: http://www.worldallergy.org.
                Accessed April 24, 2008.

6. Warner JO, Kaliner MA, Crisci CD, Del Giacco S, Frew AJ, Gh L, et al. Allergy
                practice worldwide: a report by the World Allergy Organization Specialty and
                Training Council. *Allergy Clin Immunol Int-J World Allergy Org*.
                2006;18:4-10; and *Int Arch Allergy Immunol*. 2006;139:166-174.

7. Compalati E, Penagos M, Henley K, Canonica GW. Allergy prevalence survey by the
                World Allergy Organization. *Allergy Clin Immunol Int- J World Allergy
                    Org*. 2007;19:82-90.

8. Kaliner MA, Del Giacco S, Crisci CD, Frew AJ, Liu G, Maspero J, et al; for the WAO
                Specialty and Training Council. Requirements for physician competencies in allergy:
                key clinical competencies appropriate for the care of patients with allergic or
                immunologic diseases--a position statement of the World Allergy Organization.
                    *World Allergy Organization J *[serial online]. 2008;1:42-46.
                Available at: http://www.waojournal.org. Accessed April 30, 2008.

9. Del Giacco S, Rosenwasser LJ, Crisci CD, Frew AJ, Kaliner MA, Lee B-W, et al. What
                is an allergist? A position statement of the WAO Specialty and Training Council.
                    *World Allergy Organization J *[serial online]. 2008;1:19-20.
                Available at: http://www.waojournal.org. Accessed April 30, 2008.

10. World Allergy Organization. GLORIA presentation materials. Available at:
                    http://www.worldallergy.org/educational_programs/gloria/international/materials.php.
                Accessed April 24, 2008.

11. World Allergy Organization Journal. Home. Available at: waojournal.org. Accessed
                April 24, 2008.

12. Canonica GW, Baena-Cagnani CE, Bousquet J, Bousquet PJ, Lockey RF, et al.
                Recommendations for standardization of clinical trials with allergen specific
                immunotherapy for respiratory allergy: a statement of a World Allergy Organization
                taskforce. *Allergy*. 2007; 62:317-324.

## Future Trends In Allergy Prevalence And Treatment: What To Expect?

This section contains a bibliography of general references. Further resources and
                references are provided through links from the reference list to WAO's online
                learning materials.

How will environmental and social changes influence the prevalence of allergic
                diseases in the global population, and how will the world need to respond?

## Factors Affecting Allergy Prevalence And Treatment

Although the future is difficult to predict, one thing is certain: change is the only
                "constant." Several factors will affect the future prevalence and treatment of
                allergy, including climate change, an increase in industrialization, and the
                resultant increase in exposure to allergens. A consequence of the anticipated
                increase in allergic diseases is that the present inadequacy in the training of
                physicians in the diagnosis and treatment of allergy and asthma must be
                addressed.

### Climate Change

Recently, much attention has focused on climate change and the impact of climate
                    on allergy. Experts from the World Health Organization discussed the impact of
                    climate on allergy in May 2007, and numerous articles on this topic have
                    appeared in the literature and on the Internet.^1-19 ^Climate affects
                    many aspects of allergy and allergen exposure, including the type and frequency
                    of allergens in any particular geographic location, exposure to food and insect
                    allergens, cross-reactivity among allergens, and the prevalence of
                    allergy-related diseases.

The world is in the initial stages of a global climate change that will alter
                    flora and fauna everywhere, creating changes in the profile of local allergens
                    and potentially lengthening pollen allergy seasons. As temperatures increase,
                    plant species are moving into previously temperate regions and to higher
                    elevations; similarly, when temperatures decrease, these same plant species may
                    recede back to the original habitat, whereas other plant species that typically
                    grow only in colder locales or at higher elevations may spread farther into
                    previously warmer areas. As warm seasons lengthen, so will many plant
                    pollination seasons; therefore, human exposure to allergenic pollens will
                    increase. Increases in temperature will result in increases in humidity in many
                    places, which in turn will provide a better environment for mold growth and an
                    increase in the number of airborne mold spores. Increases in temperature in
                    desert regions are reducing the amount of fertile land available for
                    agriculture, which leads to the migration of large numbers of workers into urban
                    areas. This rural to urban migration has already been shown to result in an
                    increase in the number of patients with asthma and allergy symptoms. Changes in
                    climate will affect crop patterns and increase the likelihood of new inhalant
                    allergens being introduced into a locality. In addition, insect species will
                    continue to migrate to new locations as temperatures increase. Allergies to
                    stinging insects may increase as new insects are introduced into the
                    environment, and the number of cockroaches and house-dust mites--and thus, the
                    allergen load--will increase in accordance with increases in heat and
                    humidity.

Cross-reactivity of allergens currently in the environment with new allergens
                    that are introduced through plant and insect migration could result in the
                    development of multiple immunoglobulin E (IgE) antibodies against these
                    allergens. This in turn will cause patients to react to more allergens, increase
                    the number of allergy patients, and lead to the development of more complex
                    manifestations of allergic disease.

Although attempts to reduce pollution are gaining importance in some parts of the
                    world, pollution is dramatically increasing in other developing areas. To
                    confound this issue, measures aimed at reducing atmospheric pollution will
                    likely result in a decrease in the number of patients with chronic respiratory
                    diseases, such as bronchitis. However, in areas such as the former East Germany,
                    the number of patients with allergy-related respiratory diseases reportedly
                    increased as chronic respiratory diseases decreased.

Allergy has been called "the 21st century disease" because as people live
                    cleaner, healthier, less infection-prone lives, allergies increase in frequency.
                    A theory on how improved hygiene leads to a shift in immune responses favoring
                    an allergic-type response has strong scientific support. Thus, as the world
                    develops to a more industrialized less agriculturally based economy, allergies
                    will continue to increase and plague those very areas where lifestyles are
                    improving.

### Dietary Changes Caused by Industrialization

Allergy clinicians are seeing an epidemic of food allergies in developed
                        countries.^20,21 ^Up to 10% of children in the United States and
                    Europe will develop food allergies during the first decade of life, with the
                    highest incidence in the first one or two years of life. A reduction in
                    breast-feeding by working mothers has led to an increase in exposure at an early
                    age to foreign proteins such as those in cow's milk. Moreover, these bottle-fed
                    babies are ingesting foreign proteins that would ordinarily be broken down by
                    the mother's digestive and immune system during the formation of breast milk.
                    Greater use of processed foods and preservatives may also increase the exposure
                    to more allergenic proteins. Genetic modification of food can introduce an
                    allergen into a previously "safe" food, rendering the food allergenic to those
                    who are sensitized to the modifying protein.

### Decreases in the Number of Trained Physicians for the Projected Increase in
                    Allergy Patients

Estimates indicate that up to 20% to 25% of the population of every developed
                    nation will develop some form of allergic disease.^22,23 ^As countries
                    develop more comfortable lifestyles and life-threatening infections are
                    eliminated, allergies not only will continue to increase, but will affect this
                    increasing segment of the population for a lifetime and lead to increased losses
                    in productivity both at school and in the workforce, and thereby result in
                    greater costs to society. With further reductions in breast-feeding and
                    increases in exposure to processed food, food allergies will become more serious
                    and more widespread. Accurate diagnosis and treatment of IgE-mediated food
                    allergy will become ever more important. As the Third World moves to the First
                    World and as the prevalence of allergy increases globally, the need for more
                    allergy practitioners and new allergy treatments will increase.

Clearly, there are not enough allergists to diagnose and treat the projected
                    increased number of patients with allergy.^24 ^This shortage of
                    appropriately trained health care providers is compounded by the fact that in
                    many highly industrialized countries, the overall rising costs of health care
                    have dictated a reduction in the specialty care provided by allergists, with the
                    expectation that allergy can be treated adequately by family practitioners. Many
                    patients with allergies are treated by physicians who have had an extremely
                    limited exposure to allergy diagnosis and treatment during medical school and
                    postgraduate training.

The WAO, speaking for all 77 allergy and immunology member societies worldwide,
                    has just published two position statements addressing these issues: the first,
                    "What Is an Allergist,"^25 ^defines the special clinical skill sets of
                    a trained allergist; the second, "Requirements for Physician Competencies in
                    Allergy: Key Clinical Competencies Appropriate for the Care of Patients With
                    Allergic or Immunologic Diseases,"^26 ^delineates exactly what training
                    is required for physicians at all levels to care competently for allergy
                    patients. The WAO Specialty and Training Council is now developing a recommended
                    training syllabus in Allergy and Clinical Immunology to provide a standardized
                    basic curriculum that can be adapted for local use around the world. The hope is
                    that the governing bodies of the world's medical communities will attend to
                    these position statements and not only recognize the current and future
                    deficiencies in the numbers of well-trained allergists, but also encourage
                    specialty training in allergy diagnosis and treatment on a more local level. New
                    WAO initiatives will address the importance of including allergy and clinical
                    immunology in undergraduate medical training and of the special skill sets
                    required for physicians treating children with allergic diseases.

## Changes In Allergy Treatments

### Antihistamines

Oral antihistamines have traditionally been the first-line therapy for
                    rhinoconjunctivitis and allergic skin conditions. Histamine is at least partly
                    responsible for most or all of the primary symptoms of allergy, both in the
                    mucosa of the nose and eyes and in the skin. Countless patients worldwide have
                    benefited from the use of oral antihistamines, which reduce sneezing, itching,
                    and rhinorrhea. In recent years, newer non-sedating antihistamines have become
                    available and are recommended because these agents cause less sedation, have
                    more selective mechanisms of action, and are longer lasting. Because oral
                    antihistamines are accepted and are available worldwide, these drugs will likely
                    remain the standard treatment of choice for allergic diseases, and the use of
                    nonsedating antihistamines will likely increase. Nasal antihistamine sprays have
                    also become available more recently and are very effective. In the future, both
                    oral and nasal antihistamines will likely be used to treat rhinitis, and oral
                    antihistamines will likely be used to treat cutaneous
                    disorders.^27,28^

### Corticosteroids

Recognition that topical corticosteroids are the most effective treatments
                    available for rhinitis and asthma should lead to an increase in the use of these
                    drugs for the treatment of these diseases worldwide. Some of the mature
                    corticosteroid products are very inexpensive; therefore, well-trained
                    practitioners can prescribe these modern medicines even in regions where
                    resources are limited.

The WAO anticipates that the emphasis will likely be to use more topical rather
                    than systemic treatments for rhinitis and asthma. The combination of topical
                    nasal corticosteroids and antihistamines actually potentiates the activity of
                    each drug in the treatment of rhinitis, and such a combination product, once
                    marketed as a dual-acting nasal spray, is likely be the preferred choice for
                    treating both allergic and nonallergic rhinitis. Surgical treatment of sinusitis
                    will inevitably become less popular as the understanding of this condition
                    increases and effective treatments, including corticosteroids developed in
                    vehicles that allow instillation within the sinuses, become more widely
                        available.^29-31^

Predictably, the medications currently used to treat allergy may not all be used
                    in the future. Many modern medications are cost prohibitive and are therefore
                    unavailable to much of the world. Future advances in pharmaceuticals may provide
                    relatively inexpensive treatments, which will be particularly cost effective if
                    used appropriately by both clinicians and patients.

### Immunomodulation

Modulation of the immune system to decrease the production of IgE, and thereby
                    reduce the incidence of allergic disease, should become a reality. In contrast,
                    immunomodulation to increase the production of IgG might help prevent infectious
                    diseases in patients with IgG deficiencies.

### Allergen Immunotherapy

Allergen immunotherapy has become highly standardized as allergens have become
                    characterized, which ensures an accurate diagnosis and treatment with the
                    specific allergen to which patients are sensitized.^32,33 ^Comparative
                    data indicate that allergen immunotherapy is a potent long-term treatment
                    capable of reducing the severity of allergic rhinitis and asthma. Data indicate
                    that the use of immunotherapy to treat children with allergic rhinitis reduces
                    the subsequent development of asthma. Thus, allergen immunotherapy should be
                    considered for the long-term management of appropriately selected allergic
                    patients.

Traditionally, allergen immunotherapy has been administered via subcutaneous
                    injection. Support for this preferred route of administration is based on 100
                    years of experience and the results of a multitude of studies on the use of
                    high-dose subcutaneous injection therapy. An alternative form of immunotherapy
                    is the sublingual administration of allergen, the effectiveness of which has
                    been demonstrated in European patients with rhinitis caused by certain
                    allergens, such as grass and house-dust mite. Sublingual immunotherapy has
                    become the preferred treatment of some allergic patients in Europe. Studies
                    examining the efficacy of this treatment in other populations are underway.
                    Because sublingual immunotherapy is self-administered, a strong likelihood
                    exists that this therapy will be preferred by patients once proven and approved
                    throughout the world.

Immunotherapy is the only current treatment that has long-term benefits, that is,
                    effects that last beyond the period of active treatment. Studies have clearly
                    shown that effective allergen immunotherapy can have protective effects that
                    last for years after treatment has ended. Thus, immunotherapy can reduce
                    allergic rhinoconjunctivitis and asthma, can reduce the development of asthma in
                    treated patients, can produce effects that last for years after the completion
                    of a successful program, and may become available as sublingual drops for some
                    patents. An increased use of immunotherapy in the future seems likely. However,
                    well-trained practitioners are essential to ensure the accurate identification
                    of the causative allergen or allergens, to determine when sublingual
                    immunotherapy should be used, to ensure patient safety, and to select allergens
                    that are appropriate to the patient.

### Biotechnology

Monoclonal antibodies against IgE and the various cytokines and mediators
                    involved in the allergic cascade are being extensively researched.^34-36
                    ^A humanized monoclonal antibody against IgE is already available for use
                    in adults and adolescents with moderate to severe allergic asthma and may soon
                    be indicated for use in children. Studies are underway to determine the full
                    range of atopic conditions for which monoclonal anti-IgE therapy may be
                    efficacious. Cost considerations and the availability of trained allergists to
                    determine the patients for whom this type of therapy will be effective will
                    initially determine the global availability of this class of treatments.

### Epinephrine

Anaphylaxis is an acute life-threatening condition that affects about 1% of the
                    population of most developed countries. More than 200 patients are estimated to
                    die of anaphylaxis in the United States each year. Autoinjected epinephrine
                    (adrenaline) is an extremely effective treatment of acute anaphylaxis because
                    this drug reverses all of the life-threatening processes involved in a matter of
                        minutes.^37,38 ^Practitioners in countries where this therapy is
                    licensed can readily provide epinephrine-containing devices to patients at risk
                    for anaphylaxis from stinging insects, food allergies, and other causes of
                    anaphylaxis.

The fact that many countries still do not license epinephrine injectors for
                    prescription purposes is amazing. The WAO's leadership believes that
                    autoinjectors of epinephrine need to become more widely available and that
                    physicians need to be instructed in how and when to use these injectors for the
                    treatment of anaphylaxis. In countries where epinephrine autoinjectors are
                    available, anaphylaxis can be successfully managed by patients who are trained
                    to use these devices correctly. However, epinephrine injectors should be
                    prescribed only by practitioners with the knowledge to: accurately diagnose the
                    symptoms of anaphylaxis, diagnose the causative allergens, advise patients on
                    strategies to avoid the offending allergen, and train patients in the proper use
                    of this life-saving treatment. Recognizing, even in 2007, that self-injectable
                    epinephrine devices are still not available in many countries with huge
                    populations, WAO will continue to campaign for the global availability of this
                    essential therapy.

## Disease Management Guidelines

As the incidence and importance of asthma and other allergies increase around the
                world, treatment guidelines developed in conjunction with WAO member societies will
                become even more important.^29,39 ^These guidelines will provide a broad
                framework for treatment that can be used in all communities once suitable treatments
                become widely available.

## Conclusions

The prevalence of all types of allergic diseases, such as allergic
                rhinoconjunctivitis, asthma, sinusitis, food allergies, and anaphylaxis is
                increasing. Health care resources are not increasing commensurately, and the number
                of physicians trained to diagnose and treat allergic diseases is insufficient.
                Resources can be maximized by increasing the number of physicians trained to treat
                allergic diseases and by implementing and encouraging compliance with guidelines for
                the treatment of these diseases. The need for new allergy medications, new forms of
                treatment delivery, and worldwide access to licensed allergy medications is
                urgent.
